# Dynamic capabilities in times of economic crisis. A vision of success in international markets

**DOI:** 10.1016/j.heliyon.2023.e22703

**Published:** 2023-11-22

**Authors:** Pablo Ledesma-Chaves, Jorge Arenas-Gaitán

**Affiliations:** Department of Business Administration and Marketing, University of Seville, Spain

**Keywords:** Dynamic capabilities, Export performance, Crisis, Marketing, Innovation, Learning

## Abstract

The aim of this paper is to determine the degree of influence and mediation of dynamic marketing, innovation and learning capabilities on the international performance of companies during economic crises. The analysis is developed through structured surveys of a multisectoral sample of 145 Spanish companies, using a consistent PLS approach (PLSc) for its development. The dynamic approach is developed through a methodology based on a dual time perspective in the firms' response, capturing the variation produced in the data. Dynamic marketing capabilities emerge as the most important variable, both for its direct influence on firm performance, as well as for its mediating power concerning the rest of the dynamic capabilities, which are not directly significant regarding performance. Therefore, we observe a joint effect, both direct and indirect, on dynamic marketing capabilities. The research contributes to the research field by introducing the management of dynamic capabilities and their relationships in an environment scarcely analyzed in this sense, such as that of a deep economic recession, thus helping the development of future theoretical frameworks within the Theory of Dynamic Capabilities and improving business decision-making regarding the reorganization of factors, raising awareness of the need to be oriented toward a culture of dynamic capabilities, as well as toward a correct balance in the management of resources determined by the model's capabilities.

## 1 Introduction

Since the end of the twentieth century, numerous recurrent periods of recessions and economic crises have occurred in different parts of the world: 1980–1982, 1990–1999, 2000–2002, 2008–2016, or the one we are currently immersed in due to the global coronavirus pandemic in 2020 and 2021. These are cyclical events, although not exactly foreseen, and for different reasons these intervals of years modify the perception and behavior of both consumers and managers. This change in behavior influences the way companies face and make their decisions, and above all how they shape and use their resources to solve the problems arising from a drop in demand. It is important to note that, although there are important processes of the destruction of companies, there is also a process of creation to improve their results [1]. We are in a post-COVID-19 environment as well, where we have gone through precisely an unprecedented destructive period, but where we have at the same time observed the survival of companies in international markets [2], and theoretical frameworks do not provide an adequate response. This lack of answers is not exclusive to this latest and current economic crisis, but companies are deprived of the mechanisms that theories provide, in times of prosperity, to deal with these difficult times, since theories do not contemplate variations in the environment of such a magnitude.

Internationalization is often a reaction, a somewhat desperate response when our immediate environment does not respond as it has been doing, and we are forced to look abroad for what we cannot maintain in the domestic market, using the available resources in the best possible way. It is in this situation that dynamic capabilities allow companies to advance in international markets [3]. This leads us to propose a new vision oriented toward the company's own resources and capabilities, current and future, thanks to its development capacity, and is seen as a more convenient orientation in order to achieve the objectives, especially in a situation of economic recession. Dynamic capabilities can represent that process of adaptation and reconfiguration and it is necessary to shift the point of view from the product to the capabilities [4], and turn the strategy of creating dynamic capabilities into a structural issue for companies, aligning resources with respect to the changing environment [5].

The general objective of this paper is to analyze the role of dynamic capabilities (learning, innovation and marketing capabilities concerning international performance), as well as the relationships between these capabilities. In other words, we analyzed managers' perceptions of the evolution of their learning, innovation and marketing capabilities during the crisis. At such times, companies must continue to make decisions in an environment of maximum uncertainty, which involves a process of learning and improvement in many ways. Logically, this evolution of their capabilities impacts on export performance.

To address this general objective, we develop a set of operational objectives. The first is to establish an explanatory framework for export performance through the dynamic capabilities of marketing, innovation and learning. To this end, we propose a model of relationships in which the three capabilities coexist at the same time. Second, we analyze the role of each of these dynamic capabilities in their contribution to export performance. With that in mind, an analysis of the mediation between capabilities has been carried out. Third, we analyze the relative importance of each dynamic capability. To achieve this, the total direct and indirect influence of each dynamic capability considered on export performance will be analyzed.

Our work makes several contributions. First, the evolution of dynamic capabilities in times of crisis has been little studied [6]. Secondly, to date hardly any studies have been developed on multiple capabilities and their interrelation and the mediation among them [7]. Another important academic contribution is established by contemplating internationalization within a general process of economic crisis, where several lines of research also converge, such as economic crisis processes, performance, export performance dynamic capabilities and internationalization.

The academic contributions come from the analyzed environment and the application of a theoretical framework of several dynamic capabilities, establishing the degree of interrelation and importance regarding performance. From the management point of view, the need for the implementation of a culture of dynamic capabilities is contemplated, in addition to allowing a better organization of resources in times of economic crisis. Socially, company internationalization programs should give more importance to the analysis of company capabilities, which would allow a better distribution of resources dedicated by governments to the internationalization of their companies.

## 2 Conceptual framework

### 2.1 Internationalization and economic recession

We define "recession" or "crisis" as "a process of declining demand for raw materials, products and services" [8] or as "a state in which the demand for a product is lower than at its previous level" [9]. During these periods, companies face new challenges, which differ from their behavior during economic boom periods. Their limited resources do not allow them to maintain their competitive advantage, resulting in high levels of destruction of business fabric, and with it employment, although companies try to maintain their level of activity despite having fewer markets and customers [10]. Current events have caused these definitions to be rethought, as we are facing periods of recession caused by phenomena not contemplated, such as the global pandemic that began in 2020. This has even led to the restriction of fundamental rights, including work, causing hitherto unknown effects on consumers and companies. These effects force to a greater extent the need to review how existing theories offer solutions in these periods. This study focuses on a sample of Spanish firms during the 2008–2016 global crisis. Our proposal also considers that we can gain valuable insights from the previous crisis to apply to the current crisis caused by COVID-19.

Internationalization in periods of economic crisis does not always have a negative component. Despite the fact that many companies face the process more out of obligation than conviction, they are able to compete and survive in international markets that are little known and dominated by companies of a larger size or presence. Regarding the type of company most affected, small companies have always been seen as the biggest victims of these processes, given their limited resources which do not allow them to create a sufficient competitive advantage, in addition to the difficulty in maintaining their level of activity given the restricted number of markets and customers [11]. However, it is true that not all companies are affected in the same way, and that certain companies maintain their level of activity and their competitive position, or even strengthen and prosper in such circumstances, while others go bankrupt or disappear. This makes it possible to observe a period of economic crisis as a period of creative destruction [11,12], provided that we have an understanding and ability to know the mechanisms that can give rise to such a process. The management of these crises and their consequences, are considered by factors such as overconfidence [13], gaps of knowledge and experience, a lack of independent thinking, an underestimation or absence of knowledge, producing an important process of adaptation to environmental changes, not always successfully, and where innovative management and the creation of new value becomes a fundamental factor [14]. The question is what tools or processes we can use to transform such a negative period into an opportunity.

### 2.2 Dynamic capabilities

Research on dynamic capabilities is among the most promising approaches within the strategic research agenda [15,16]. In fact, dynamic capabilities make a contribution on a double track. On the one hand, they are the basis for the introduction of valuable and unique novelties in the resource base, and, on the other hand, they are a tool for the continuous promotion and reconfiguration of existing resources and capabilities [17]. Companies have changed the orientation of their priorities through the dynamic capabilities influenced by the pandemic period, having to apply new strategic orientations for their continuity, such as resilience, creativity or the effective development of capabilities [18]. By combining evolutionary and resource-based logics, the dynamic capabilities view focuses on capabilities that can confer a competitive advantage through adding unique value to the firm through systematic changes [19], which becomes a source of competitive advantage and performance improvement [20]. Given the time that it has been developing, we can consider dynamic capabilities as an emerging field, so current research is beginning to offer concepts, definitions and models, as well as advancing the different research paths to be followed [21].

We start from the definition of dynamic capabilities as *" … the firm's potential to systematically solve problems, formed by its propensity to sense opportunities and threats, to make timely and market-oriented decisions, and to change its resource base."* [22]. Previous research has established the critical role of dynamic capabilities in survival in complex firm environments [23,24]. In the present investigation, there are three dynamic capabilities analyzed and proposed in the model: Learning, innovation and marketing, which are among the most analyzed in the literature [4], although their joint analysis has not been proposed so far [25].

### 2.3 Dynamic learning capabilities

Dynamic learning capabilities are defined as the combination of practices that promote learning within the organization among employees, collaborative arrangements with other organizations that enable learning to be extended, and an open culture within the organization that promotes and sustains knowledge sharing [26]. Learning capabilities have been related to the emergence of new business solutions in environments of economic crisis [27]. The generation of continuous organizational learning allows the development of skills that affect decision-making and the establishment of routines in times of great difficulty. [28]. It has a significant impact on organizational effectiveness and performance, as it encompasses the management practices, mechanisms and organizational structures that can be implemented and, in addition, involves a more or less permanent change in behavior, aimed at achieving organizational effectiveness [29]. Dynamic learning capabilities represent the origin of its strategic base [30], and allow the development of the rest of the elements, as well as endowing them with the understanding of the surrounding environment, its interpretation and cohesion in continuous decision-making in the dynamic process confronted in the face of turbulent international markets. Our model considers dynamic learning capabilities as a meta-capacity which entails a strategic redirection of the company when up against change.

### 2.4 Dynamic innovation capabilities

Dynamic innovation capabilities are the ability of an organization to present and develop new ideas to the market, leading to competitive advantages and profit improvements in the short or long term [31]. Innovation capabilities are considered a source of competitive advantage [32]. that can determine the company's survival in a generalized crisis environment [33]. In order to be innovative and achieve sustainable competitive advantages, firms must develop innovation capabilities [7,34]. These are an internal capability, a "potential to produce innovations", and therefore require continuous improvements in order to produce the necessary value [35], and for new creations to be more effective than those of one's competitors [36]. The importance of innovativeness derives from the fact that it contributes to the dynamic competitive advantage of firms, as it enhances their ability to keep up with, respond to and initiate technological changes on a continuous basis [37].

### 2.5 Dynamic marketing capabilities

Dynamic marketing capabilities are an important strategic capability for firms to develop because they are closely associated with several aspects, such as the ability to supply products effectively [38], meet customer needs, and support the leveraging of other advantages through market sensing and customer linkage [39]. The basic role of dynamic marketing capabilities in the creation of sustainable competitive advantages in complex environments has been analyzed by [40]. From a foreign market point of view, dynamic marketing capabilities can also play a key role in improving the performance of multinational companies seeking collaborative entry modes, such as international joint ventures, alliances, and acquisitions [39]. Marketing capabilities are understood as the ability to create and share superior customer value through an efficient and rapid response of marketing processes. This has been widely suggested as one of the critical factors contributing to firm performance and sustaining competitive advantage [15,[41], [42], [43]]. The introduction of new methods and innovations through marketing capabilities is reflected in studies such as that of Wang et al. (2020), which analyzes the survival of Chinese companies by implementing innovative marketing strategies via dynamic capabilities. Also, [44] analyze the relationship between marketing and innovation capabilities for competitive advantage from a dynamic capabilities perspective.

### 2.6 Hypotheses

**Fig. 1:**
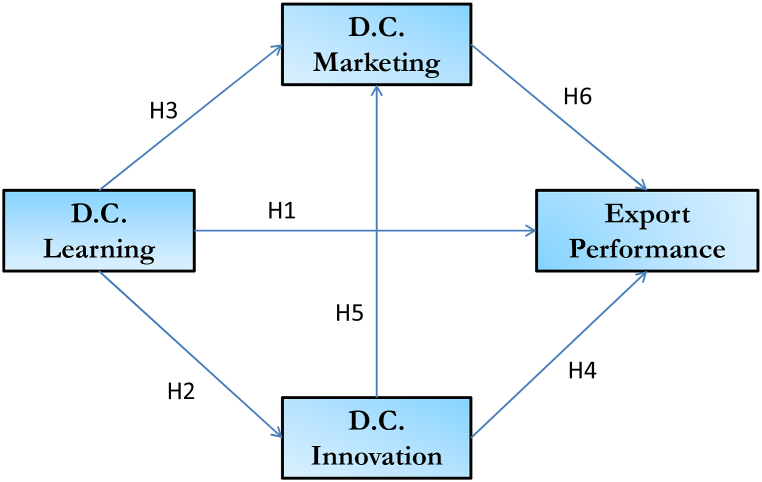
Proposed model.

The proposed model can be seen in Fig. 1. Our work proposes a direct relationship between dynamic learning capacity and international performance. The dynamic vision of learning, understood as a capacity, is manifested in the ability to generate new knowledge within an organization, which in most cases is unique, through the constant creation, incorporation and remodeling of its fundamental skills, including the ability to acquire or maintain competitive advantages [45]. The company, through its adaptive ability to continuously learn, demonstrates its capacity to adjust and acquire knowledge in constantly changing environments. [46], implementing practices that are more effective, efficient, and innovative [47].The literature considers that learning itself becomes an essential element for improving competitiveness [[48], [49], [50]], and that it transforms behavior by reflecting new knowledge and ideas [51]. There is also evidence from empirical studies that directly relate learning ability to positive entrepreneurial outcomes [[52], [53], [54], [55], [56]]. Given the above, we propose the hypothesis.

H1 Improvements in dynamic learning capabilities lead to a better international performance of exporting firms in periods of economic downturn.

Learning-oriented organizations are found to be more apt for the assimilation of new ideas [57]. Dynamic learning capabilities are a complex process with several phases and which are closely related to innovative performance [58] and are a key factor in organizations that wish to grow and innovate [59], also having a positive effect on the innovativeness of firms [50,60]. The link between dynamic capabilities and performance, as well as with innovativeness, is realized through knowledge management and learning [[61], [62], [63]]. Even though the empirical literature is scarce, there are some papers that find a direct relationship between innovation and learning [50,57,64,65], as well as the existence of studies that determine that dynamic capabilities positively affect performance [66]. For all these reasons, we propose the following hypothesis.

H2 Improvements in dynamic learning capabilities lead to improvements in dynamic innovation capabilities in exporting firms in periods of economic crisis.

The application of learning and knowledge to marketing problems already appears in the work of [67]. [68] analyze the influence of learning orientation on the firm's marketing capabilities [69]. develop 14 marketing skills that are enhanced by prior learning processes [70]. positively test the hypothesis that market-oriented learning has a positive and significant effect on the firm's marketing capabilities. Other, more recent, studies also confirm the relationship between learning capabilities and marketing capabilities [[71], [72], [73]] [[71], [72], [73]] [[71], [72], [73]]. Firms with a strong market-oriented learning capability collect information about changes in the market, and this knowledge facilitates the effective use of marketing strategies. In the case of economic recession, the information obtained from the market through learning processes is crucial for the company and is to be translated into effective market strategies through dynamic marketing capabilities. Therefore, we consider that.

H3 Improvements in dynamic learning capabilities lead to better results in the dynamic marketing capabilities of exporting firms in periods of economic downturn.

The presence of innovation as a dynamic capability within the company shows the existence of skills and tools as well as ways of acting that grant a competitive advantage in the form of advantaged market positions over one's competitors [74]. A dynamic conceptualization of innovation as a capability implies an intensive and deep search among the different sources of external knowledge [75]. It demonstrates the ability to generate new ideas, discover new possibilities in the market and apply novelties by taking advantage of available resources and skills. In other words, a dynamic innovative capacity refers to the constant ability to convert knowledge and concepts into completely new products, processes and systems that benefit the company [76], in a continuous process of adaptation to the environment and new circumstances. Innovation becomes one of the key factors in improving the international performance of companies [[77], [78], [79], [80]]. Another characteristic that innovation confers is market power, and it therefore facilitates export activities [81]. The adoption of dynamic innovation capabilities positively influences export strategy [82]. Innovative firms are better prepared to operate in an unstable environment, such as an economic crisis [50]. A firm with a high capability to learn improves its export performance [54,56,83]. Therefore, we establish.

H4 Improvements in dynamic innovativeness lead to a better international performance of exporting firms in periods of economic crisis.

Dynamic marketing capabilities play a key role in the development of innovation capabilities, and in the firm's own development, as they introduce innovations at the right place and at the right time to achieve competitive advantage [84]. Sufficient marketing skills and resources are necessary for the development of innovations [85]. The complementarity of marketing and innovation is critical to firm performance. The generation of knowledge about the market involves a collective effort among all the company's areas, especially in innovation [86], and marketing capabilities are able to transform it, in times of stress in the environment, into market knowledge, competitive advantage, and positive results for the company. Therefore.

H5 Improvements in dynamic innovation capabilities lead to better results in the dynamic marketing capability of exporting firms in periods of economic crisis.

Dynamic marketing capabilities are based on market knowledge, which can distinguish between successful and unsuccessful firms in international environments [38,87,88]. The dynamic process in this capacity is seen in a proactive orientation toward the changing market, as well as the development and market availability of new products on a continuous basis [89]. In companies that have it, knowledge in this area is fundamental, and it becomes a permanent tool for absorbing market knowledge [90]. In addition, there is a continuous dissemination of market knowledge within the organization [91], involving cross-functional coordination across the entire organization [92].

Dynamic marketing capabilities enable firms to build profitable customer relationships and maintain the already achieved customer base, which in turn impacts organizational performance [93]. Dynamic marketing capabilities affect performance by generating satisfaction and loyalty and superior market performance [94], and firms can use them to be better positioned, launch better products, adjust their pricing system, distribute more effectively, and tighten their customer relationship [95,96], improving their performance [97,98], and adapting swiftly to changes [39,41]. In other words, increasing dynamic marketing capabilities can lead to improving the firm's domestic and international performance. In this regard, we formulate the following hypothesis.

H6 Improvements in dynamic marketing capabilities lead to a better international performance of exporting firms in periods of economic downturn.

## 3 Methodology

### 3.1 Sample

The basis of the study is a multisectoral sample of Spanish companies from the Andalusian Agency for Foreign Promotion (EXTENDA) and the Industrial and Exporting Companies Catalog of the Basque Country (CIVEX). A total of 750 questionnaires were sent to the heads of the internationalization departments and general managers of the companies, and 145 valid questionnaires were reported, representing a response rate of 19.3 %. This result is within the acceptable range of 15–20 % (Menon et al., 1996). The consideration of a multisectoral sample widens the observed variance (Table 1), thus emphasizing the generalizability of the results [99]. A pretest was carried out with several experts to ensure the correct interpretation of the items. The questionnaire was conducted in Spanish. Prior to its use, it was translated from English into Spanish and then back into English to ensure the accuracy of the translation [100]. The presentation of the questionnaire included an agreement regarding the informed consent of all the research participants prior to the start of the research.

**Table 1:** Sample characteristics.

Sector distribution	
Agri-food	**31 %**
Industrial	**32 %**
Services	**37 %**
Number of employees	
1–11	**37 %**
11–49	**38 %**
50–249	**18 %**
More than 250	**7 %**
Export dedication	
Has staff dedicated	**53 %**
Personnel from other areas deal with export	**47 %**
Presence of export department	
Has its own export department	**43 %**
Integrates export in other departments	**57 %**
Start of export activity	
Same year of starting the activity	**11 %**
Within the first 5 years	**45.5 %**
From the 6th year of activity onward	**54,5 %**
Number of countries exported to	
To 10 or less countries	**26 %**
To more than 10 countries	**74 %**

The sample size, 145 cases, is in line with previous research. However, we previously conducted an analysis in accordance with the recommendations of Faul et al. (2007), where it is specified that the sample size to reach acceptable levels of statistical power must be set according to the effect size (f^2), the power or probability of detecting an effect in the sample that actually exists in the population; and the Alpha, as the probability of false positives, statistical significance appearing in the sample when in fact it is not present. All of this also taking into account the number of predictors, or the largest number of structural paths directed to a latent dependent variable, there being in our case 3 which affect the Export Performance.

By consulting the tables produced by Green (1991) for an advanced power of 0.8, an average effect size (f^2^) (0.15) and an alpha of 5 %, as generally accepted measures [101] for social research, would yield a sample size of 43. We checked this by entering the data into the G*Power program. G*Power is a free software program used to calculate statistical power, which includes most statistical tests and is widely used in all types of scientific fields as well as in the social sciences [102,103]. This sample size is well below that obtained by us, 145, so we consider it to be an adequate sample size, representative of the total population, and whose conclusions can be generalized with a high level of statistical probability.

### 3.2 Scales of measurement

One of the main contributions of this work lies in the way of addressing the evolutionary nature of the variables, which is especially important for dynamic capabilities. Our study asks respondents a question that they must answer by placing themselves in two different temporal moments, establishing the recent economic crisis as a temporal or differential milestone of the response [[104], [105], [106]]. That is, they must answer the same question from the perspective of "before the crisis" and "after the crisis". This way of analyzing creates a differential variable between both answers. We take the idea of episodes from Ref. [105] and have applied it to the crisis like [106] and following [104] who ask about export performance before and after the crisis. This technique has received academic support in other works [15].

The literature review identified the measurement scales for each of the constructs analyzed. The dynamic innovation capability and dynamic marketing capability variables are evaluated by means of the [107] scale, and the dynamic learning capability variable through the [108] scale. Likewise, the variable "International performance" is measured using the [109] scale. This measure of business performance consists of five dimensions, and is specially adapted for international business and has also been widely used [67,[110], [111], [112]]. A 7-point Likert scale was used to measure the variables.

### 3.3 Statistical tools

We used PLS analysis to test the proposed research model. PLS is a variance-based structural equation modeling technique that is widely applied in business and social science research [113]. However, PLS-SEM has shown inconsistencies in the estimation of path coefficients in the case of reflective measures, which has adverse consequences for hypothesis testing [114]. Although some authors have pointed out that the use of PLS and PLSc can be complementary, this is not always possible [115]. The choice of PLSc avoids inflated loads and brings consistency to path coefficients [116]. In addition, in the case of reflective models, PLSc provides goodness-of-fit better than traditional PLS [117] and does not produce the downward estimate of R-squared reported by the latter [118]. Researchers such as [119] propose their application for simple and highly reliable models. Therefore, in this case we have chosen to use consistent PLS (PLSc) in this work.

## 4 Results

Following the lines drawn by the analysis with PLSc, we first analyzed the reliability and validity of the scales, and then assessed the proposed structural model. Finally, we performed an analysis of the relationships within the model, interested in determining the probable existence of synergies between the capabilities.

The analysis of the reliability and validity of the measurement model followed the recommendations which appeared in the literature [113,120]. In the case of reflective variables, first of all, the individual reliability of the item is ensured. To do so, the factor loadings (loadings) on their own latent variables are examined. These loadings must be higher than the 0.7 proposed in the literature. These results are shown in Table 2.

**Table 2:** Loadings of the different items.

Dynamic innovation capabilities	
Relative to our competitors, my company is good at setting up new types of manufacturing facilities and operations	.814
Relative to our competitors, my company is good at learning about technology it has not used before	.836
Relative to our competitors, my company is good at recruiting engineers in technical areas it is not familiar with	.893
Relative to our competitors, my company is good at assessing the feasibility of new technologies.	.865
Relative to our competitors, my company is good at identifying promising new technologies.	.834
Relative to our competitors, my company is good at implementing new types of production processes	.803
**Dynamic marketing capabilities**	
Relative to our competitors, my company is good at assessing the potential of new markets.	.648
Relative to our competitors, my company is good at building relationships in new markets.	.922
Relative to our competitors, my company is good at setting up new distribution channels	.749
Relative to our competitors, my company is good at setting up a new sales force	.867
Relative to our competitors, my company is good at leveraging its brand reputation or company image to new markets	.959
Relative to our competitors, my company is good at researching new competitors and new customers.	.820
Relative to our competitors, my company is good at developing new advertising or promotion strategies	.722
Relative to our competitors, my company is good at developing new pricing strategies	.834
**Dynamic learning capabilities**	
We have a comprehensive program for employee learning	.907
We have an organization-wide training and development process, including career path planning, for all our employees	.802
Employee learning is a topic that is discussed intensively by the top management	.709
The attitude prevailing here is that employee learning in an investment, not an expense	.869
We always upgrade employees' knowledge and skills profiles	.913
Managers agree that our organization's ability to learn is the key to our competitive advantage	.843
**International performance**	
Rate your organization relative to all other competitors in your principal served market segment (PSMS) over the past year on market share	.969
Rate your organization relative to all other competitors in your principal served market segment (PSMS) over the past year on export propensity	.789
Rate your organization relative to all other competitors in your principal served market segment (PSMS) over the past year on profitability	.874
Rate your organization relative to all other competitors in your principal served market segment (PSMS) over the past year on sales	.721

We then undertook the analysis of the reliability of the constructs, for which we used composite reliability and Cronbach's alpha (Table 3). In addition, we ensured convergent validity by studying the Mean Extracted Variance. For the first set of variables, our indicators are above the 0.7 threshold, and for the AVE they are well above the 0.5 recommended by the analyses [121].

**Table 3:** Cronbach's alpha, composite reliability and AVE.

	Cronbach's alpha	rho_A	Composite reliability	Average extracted variance (AVE))
Learning	.936	.940	.936	.711
Export Performance	.909	.918	.907	.712
Innovation	.936	.936	.936	.708
Marketing	.942	.949	.942	.674

For the evaluation of discriminant validity, the research follows the criterion of Hair et al. (2021), where the use of the Heterotrait-Monotrait ratio (HTMT) is recommended [122]. This in all cases offered levels below the recommended 0.9 (Table 4). It is important to check that the HTMT values are significantly different from 1, so we report the confidence intervals. The results allow us to ensure the discriminant validity of the latent variables employed [121].

**Table 4:** Confidence intervals for HTMT ratio.

	Original Sample (O)	Sample mean (M)	2.5 %	97.5 %
**Export Performance - > Learning**	.284	.296	.140	.475
**Innovation - > Learning**	.547	.547	.324	.748
**Innovation - > Export Performance**	.412	.418	.258	.573
**Marketing - > Learning**	.623	.622	.393	.831
**Marketing - > Export Performance**	.477	.489	.322	.662
**Marketing - > Innovation**	.787	.788	.657	.888

The R^2^ value depends on the complexity of the phenomenon and the model. Our results show an explained variance value with respect to international results of almost 24 % (Table 5), which is a considerable value in this type of research [123]. The value is also representative since our model has few exogenous constructs, which with a high R^2^ value makes the model parsimonious. The low complexity of the model makes it unnecessary to resort to the adjusted coefficient of determination. It is also important to highlight the amount of variance explained about the Dynamic Marketing Capability construct, which looking at the results and interpretation becomes key to the model.

**Table 5:** Explained variance (R^2^ value).

	R^2^
**Export Performance**	.238
**Innovation**	.303
**Marketing**	.679

**Fig. 2:**
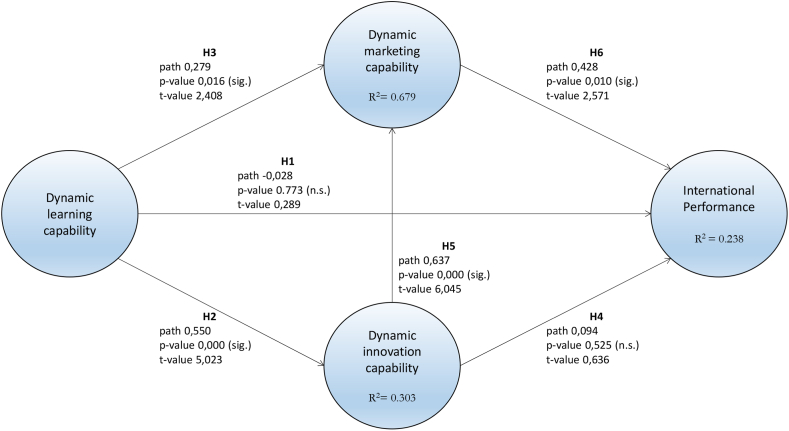
Structural model and relationship between variables.

After the evaluation of the measurement model, we will proceed with the structural model in order to test the hypotheses. The process is performed by bootstrapping 5000 subsamples, which we will use to test the statistical significance of the different paths, as well as the explained variance of the endogenous variables. This information can be seen in Fig. 2.

The goodness-of-fit is proposed through the SRMS criterion, which provides a value in our case of 0.054. This value is below the 0.08 proposed by Ref. [124], so it is considered a good fit. We also performed an analysis of the VAFs to test the indirect relationships in order to verify the complementarity of the dynamic capabilities analyzed.

Finally, and as a compilation, we evaluated the significance of the direct effects in the model, for which we used the results of the bootstrapping with 5000 subsamples performed previously, a process that we carried out to obtain the t-values as well as the percentiles of the confidence intervals [125]. Addressing the data in Table 6, hypotheses H2, H3, H5 and H6 are confirmed, having direct and significant relationships with each of their variables. For these four variables, the f^2^ levels exceed the minimum threshold of 0.02 [126]. However, the effects described by hypotheses H1 and H4 are not significant, in addition to having f^2^ values below 0.02. This situation leads us to consider that the existence in itself of certain dynamic capabilities, such as innovation and learning, as well as culture and orientation toward their creation, are not sufficient to achieve success in international markets when the environment is fraught with uncertainty in the face of an economic crisis.

**Table 6:** Effects on endogenous variables.

		Direct effect	t-value	p-value	Confidence Interval	Explained Variance	f^2^
Export Performance							
R^2^ = 0.238							
H1	CDAp→ EP	−0.028	0.289	0.773	[-0.210; 0.181] No Significant	−0.008 %	0.001
H4	CDInn→ EP	0.094	0.636	0.525	[-0.184; 0.398] No Significant	3.919 %	0.004
H6	CDMk→ EP	0.428	2.571	0.010	[0.070; 0.739] Significant	20.758 %	0.077
CD Marketing							
R^2^ = 0.679							
H3	CDAp→ CDMk	0.279	2.408	0.016	[0.092; 0.550] Significant	17.549 %	0.169
H5	CDInn→ CDMk	0.637	6.045	0.000	[0.383; 0.808] Significant	50.323 %	0.880
CD Innovation							
R^2^ = 0.303							
H2	CDAp→ CDInn	0.550	5.023	0.000	[0.329; 0.756] Significant	30.25 %	0.434

In order to analyze the mediation performed by the different variables between them, the mediation analysis follows the recommendations of Hair Jr et al. (2021), where reporting and comparison through direct, indirect, specific and total effects is indicated to detect possible mediations between constructs [127,128]. To this end, we can observe Table 7, where all the relationships that have an impact on EP are shown. The hypothesis test shows the direct significance of only one variable with respect to Export Performance, namely DMC (p-value 0.010). However, the observation of indirect effects reveals that both DIC and DMC, acting as mediating variables (in parentheses), cause the relationships between DLC and DIC to appear as significant with respect to EP (p-values 0.000 and 0.023).

**Table 7:** Mediation Analysis He puesto puntos decimales: -

			path coefficient	p-value	t-value	Significance
Direct effect	DLC- > EP		−0.028	0.773	0.289	No
	DIC- > EP		0.094	0.525	0.636	No
	DMC- > EP		0.428	0.010	2.571	Yes
Indirect effect	DLC- > EP	(DIC)	0.321	0.000	3.623	Yes
	DIC- > EP	(DMC)	0.273	0.023	2.277	Yes
	DLC- > DMC	(DIC)	0.350	0.000	4.801	Yes
Specific indirect effects	DLC - > DIC - > EP		0.052	0.551	0.597	No
	DLC - > DMC - > EP		0.119	0.075	1.782	No
	DLC - > DIC - > DMC - > EP		0.350	0.027	2.205	Yes
Total effect	DLC - > EP		0.293	0.002	3.091	Yes
	DIC - > EP		0.367	0.001	3.437	Yes
	DMC - > EP		0.428	0.010	2.571	Yes

DLC: Dynamic Learning Capabilities; DIC Dynamic Innovation Capabilities.

DMC: Dynamic Marketing Capabilities; EP: Export Performance.

The specific indirect effects reveal the existence of a significant relationship (p-value 0.027) between the three dynamic capabilities analyzed in achieving international results. This fact reinforces the idea of the interrelation and relational capacity among the variables. This means that, finally, the total effect on PE is significant for all the variables.

## 5 Discussion

The results obtained have allowed us to achieve our main objective of analyzing the role of the dynamic capabilities of learning, innovation and marketing in international performance. And we are going to go deeper into the results following the order of the secondary objectives.

The first secondary objective referred to the construction of the model of relationships between capabilities and their direct influence on performance. As for the hypotheses that directly relate dynamic capabilities to performance, no support has been found for H1, regarding dynamic learning capabilities and performance. This is contrary to the assertions of other works [129]. These authors indicate that organizations may not have adequately understood the importance of learning due to the uncertainty that they may be experiencing in such crisis environments. However, it is possible that not all organizations are prepared to share knowledge, a prerequisite for the development of dynamic learning capabilities, favoring the achievement of strategic objectives and overcoming obstacles [46]. The process comes from a double vision, where firstly organizations learn and generate knowledge from the development of dynamic learning capabilities, and subsequently activate the learning process between them [130], reverting the success of learning on better performance. However, it could be posited whether the special circumstances of the economic crisis in international companies or the lack of association between them are the causes of the lack of this direct relationship. This relationship may also have an important conditioning factor derived from the difficulties offered by the environment. Analyses such as that of [131] indicate that organizations may not have adequately understood the importance of learning due to the uncertainty they may be experiencing in these crisis environments.

Something similar occurs with H4, the hypothesis relating dynamic innovation capabilities to performance, which our results do not support, contrary to some studies [110,129,132]. Certain analyses have indicated that dynamic innovativeness capabilities exert an influence on performance whenever they lead to the development of innovation activities [133]. Studies have indicated that dynamic innovation capabilities are determinant as long as they have been previously developed through learning capabilities [132,133], but considering both elements in a differentiated manner. That is, emphasizing dynamic learning capabilities for their value of knowledge gain and dynamic innovation capabilities oriented toward the desire for organizational change [132]. The collaborative effect of innovation could also be a detriment for Spanish firms, as some analyses have indicated that the effect of innovation capabilities on performance occurs when they are developed collaboratively (Najafi-Tavani et al., 2018). The effect of the turbulence of the environment, to the extent of the uncertainty present regarding the development of dynamic innovation capabilities, could also be present in terms of not confirming the hypothesis, at least in the Spanish environment, although in other environments its influence has been only partially demonstrated, in addition to also being affected by factors such as intersectoral characteristics [134].

For H6, which relates dynamic marketing capabilities to international performance, we do obtain support. However, our work wants to stop here in particular. The analyses we have conducted have confirmed the presence of dynamic marketing capabilities in international performance both directly and through a mediating role [15,135]. Therefore, we first highlight the importance of this hypothesis for the analysis of our results, since, in one way or another, in the most complex periods of the environment, such as an economic crisis, the presence of marketing in the form of dynamic capabilities is essential for the achievement of positive results. The development of our model, its approach, indicates that, without dynamic marketing capabilities, facing the economic crisis is likely to be a failure. Learning and innovation are upstream factors converted into market developments through dynamic marketing capabilities. However, previous analyses have indicated that dynamic marketing capabilities would not have a direct effect on performance [136] but would depend on the efficiency of the resulting resources. Our analysis is in favor of this thesis once the indirect effects have been analyzed and looking at the unproven direct relationship of the other capabilities. The relationships suggest that the great development and influence achieved by dynamic marketing capabilities in times of economic crisis on international performance stems from the contribution or influence of the preceding capabilities, which allow them to play a pre-eminent role in performance contribution. The results are in line with research such as that by Dejardin et al. (2023), which analyzes the changes in corporate behavior after COVID-19 and establishes the importance and influence of dynamic marketing capabilities on performance.

Our second objective was directed toward the analysis of the relationships and mediation between the different capabilities within the model with a view to export performance, for which we analyzed the effects between the different dynamic capabilities. Despite the non-direct relationship with performance, the dynamic capability of learning is shown to be significant concerning marketing and innovation capabilities, respectively, considering hypotheses two and three. In the case of linking dynamic learning capabilities and dynamic innovation capabilities (H2), the result appears in previous analyses [50,137], bearing in mind that those companies which wish to innovate, must undergo a learning process. However, the learning developed here as a dynamic capability has a double aspect for the companies in the sample. Firstly, learning about international markets, which allows them to innovate their products and processes dynamically and quickly. And secondly, learning about the process in which they are immersed, since the arrival of the economic crisis does subject them, if not instantaneously, to an environment of uncertainty that they need to know better. It is therefore necessary to acquire new knowledge [50] for the success of dynamic innovation capabilities. Companies need to understand the changing and complex environment of an economic crisis before undertaking changes or innovations in their processes, which in the first instance remain unchanged, while waiting for a better approach to the problem. The importance of the moderation or gradual intervention of capabilities is being investigated as a relevant element of the interaction between the development of dynamic capabilities, competitive advantages and performance [75]. The dependence on the environment in such circumstances makes generalizations complicated, and the special characteristics of Spanish companies and their internationalization processes lead us to think that there may be a relationship, perhaps of prevalence or at least of a certain order, between both capabilities. It is true that among the scientific community there have also been calls to clarify this relationship, not yet correctly determined due to the very indefinition of some concepts [138].

We observed a similar result in the dynamic learning capability and its influence on marketing (H3), although the intensity of their relationship, the former being quite high based on the results, is even higher. This positive relationship is in line with previously conducted analyses [139]. The marketing problems arising from the new environmental conditions are being solved by Spanish companies through an adaptation of their competitive characteristics as the new recession environment had required this. However, even knowing that the influence and significance of dynamic learning capabilities regarding dynamic marketing capabilities is high, it is difficult to know how this relationship translates, since, according to some studies, it is hard to identify all the marketing capabilities developed by companies [140] and, therefore, to really know which ones are being enhanced through learning. The marketing capabilities analyzed, such as strategic management, communications, planning and market innovation management, are developed by companies according to their individual circumstances, taking into account the environment. Hence, Spanish companies in this period of economic crisis have noted the impact of these characteristics to obtain superior performance, although this would be worth analyzing in greater depth to determine which have been the most decisive issues.

The influence of dynamic innovation capabilities on dynamic marketing capabilities is supported by H5. Innovations can be considered as a process of different activities, i.e., responding to dynamic changes and improving existing products, services, competencies, skills, but also business models and the development of different marketing strategies, etc. Likewise, innovations can be considered as an outcome, i.e., responding to high competition and developing new products and services, technologies, creating new business models, markets, and so forth [50,141]. Jiménez-Jiménez & Sanz-Valle (2011) add that mostly in the scientific literature innovations are conceptualized as adapters of new ideas, results or behavioral changes. In the internationalization process of Spanish companies in periods of economic crisis, they have had to adapt to the circumstances by modifying their marketing strategies in all aspects, and this innovative process, first, has led to the improvement of the latter. Our analysis looks at dynamic marketing capabilities from all aspects, so we anticipate a broad improvement in the company's marketing management process and, therefore, in the development of dynamic capabilities within this area. Innovative capabilities in themselves are not necessarily the only factor driving the achievement of superior international performance. This is because, while Spanish firms in periods of economic uncertainty can leverage their innovative capabilities to develop new product offerings, it is dynamic marketing capabilities that help firms bring products to market and serve the market better than their competitors, a conclusion that is in line with [142].

Our third objective sought to determine the relative importance of each of the dynamic capabilities analyzed. To do so, we resorted to the total, direct and indirect influence of each of them. In the case of the Dynamic Marketing Capability, this was the only capability whose direct influence on performance was supported by H6, a fact that coincides with previous research [15], which also revealed the importance of the Dynamic Marketing Capability as an enhancer of Capabilities. As a result of this, our research finds the total effect of Dynamic Learning and Innovation Capabilities to be significant. It happens that the relationship of both capabilities with performance is significant whenever the Dynamic Marketing Capability intervenes, as shown by the results of our research where the results indicate a total mediation. This fact had not been sufficiently demonstrated in the current literature, representing an important advance in terms of the intervention of the Dynamic Marketing Capability as a variable with a synergic effect on the rest of the capabilities in periods of economic crisis, something that could be very important in the current circumstances of COVID-19.

## 6 Academic, managerial and social implications

### 6.1 Academic implications

From an academic perspective, our work makes several contributions to research on this topic. First of all, we have conducted this research in an environment that has been little analyzed in the field of dynamic capabilities. Periods of economic crisis are considered within the field of economic sciences as anomalous situations, when reality shows that they are more or less predictable cycles in which companies must learn to develop their capabilities if they want to survive. Therefore, our study contributes, in this sense, by placing dynamic capabilities where research had not done so far, providing alternatives to the theoretical frameworks proposed in the face of the alteration produced in the environment. Economic downturns are cyclical and therefore companies are aware that they must adapt their strategy for creating dynamic capabilities from time to time, something that our work offers outside the established analyses in times of economic normality. Secondly, most analyses to date have focused on one dynamic capability, or at most two, trying to determine their influence on some variable. This research contemplates the introduction of three capabilities, analyzing not only their direct influence on the final result but also proposing a relational model among them. So far, studies that have contemplated more than two capabilities have been very scarce, given their difficulty and constructive complication and the lack of theoretical references. In addition, among the most noteworthy results of the study is the indication of the existence of a complementarity between capabilities, i.e., a kind of order of development of these capabilities with a view to improving international performance. Thirdly, and derived from the above, although combinations of dynamic capabilities have been analyzed, there was no progress in determining which were more important. Despite the scientific community justifying this statement on the basis of the idiosyncratic nature of the development of dynamic capabilities, we can offer, within a general and recurrent framework such as that of economic crises and in a developed environment such as Spain, a starting point for determining the relative importance of each of the capabilities and their contribution to international performance. Subsequent analyses should confirm this situation, involving, if necessary, new dynamic capabilities and a reordering of these capabilities to improve the results obtained. Finally, our work establishes a dynamic approach to the measurement of the variables. For all the constructs, we determine that the respondent answers the same question at different times, as explained above. This measurement is based on previous studies of a retrospective nature and serves to solve the problems of availability and time, and above all of opportunity, since it is complex without an a posteriori vision to situate the limits of an economic recession, if not with a broader time perspective that may cause the novelty of the data to be lost. For this reason, and through this vision the respondent is in an optimal position to make a mental comparison between both temporal moments, having a greater capability for comparison and reflection on his/her situation at both moments. For our part, both responses for the same construct are used for the creation of a third differential variable that allows us to better capture the dynamic nature of the process, and thus be able to operate with the statistical instruments. The results have shown a high level of reliability, consistency and predictive capability of the model. In accordance with [143], we are not recommending an interpretation, but rather seeking the best way to measure the difference in perception between the two temporal moments and to defend the proposal. The validation of the construct is based on its ability to reflect the meaning of the set objective, as well as its use in the generation of testable and tested hypotheses [143].

### 6.2 Managerial implications

From a managerial point of view, the fundamental implication for managers is clear: it is necessary to manage and develop dynamic capabilities according to environmental conditions. The company's management must be able to become aware of its capabilities, i.e., to look inward in order to project itself outward. We understand that this process in an environment of economic crisis is complex, since the stress generated by these situations within companies is considerable, and there is a tendency toward short-term problem solving rather than a more far-reaching overall vision. However, our analysis contemplates that it is possible to go further, and therefore that the development of strategies oriented toward the creation of dynamic capabilities is compatible with processes of pressure within the environment, with enormous possibilities that the results in international markets will be positive, having created capabilities that will undoubtedly benefit the company in the future in all markets where it is present. Managers must lead the process of creating a culture of dynamic capabilities within the company. Therefore, companies can establish, within the development of a culture of dynamic capabilities, intervention mechanisms that allow them to face periods of economic crisis, as a more favorable reorganization of resources that gives them an extra preparation to face the crisis process with better guarantees and performance.

According to the model developed, the most valued and useful knowledge of managers is not only associated with a knowledge of internationalization but also with a process of learning and creativity in a global context that enables them to learn more about how the internationalization process develops. The development of dynamic capabilities is linked to the people who carry it out. This global learning will lead to innovative processes that will improve managers' decisions for better international performance. The development of dynamic capabilities does not only rest at the company level. Personal skills are at the core of the development of dynamic capabilities that create competitive advantage. Our research therefore confirms that the importance of a learning-by-innovation model through management experience can be shown to be a better way to channel and monetize foreign investments. Companies can, therefore, in a context of promoting dynamic capabilities, establish processes within the company to attract innovative talent and learning, finding those people with the greatest predisposition to solve the problems and make the decisions that occur in a changing environment of economic crisis.

Within the company's strategy, and framed in the theory of cost allocation, managers must assess very carefully how to redistribute resources in the new situation of economic crisis in the environment. If we look at the results in terms of the weight of capabilities according to our analysis, managers must consider very carefully whether it is really worth investing money in those areas where the company's potential has traditionally been higher. Let us look at an example situation: In the case of a company whose innovative processes and capabilities are highly developed, it is very likely that, in times of economic crisis in the face of market pressure, its managers will tend to boost that area as a competitive advantage in the face of international markets. In itself, their approach is not wrong: perhaps the dynamics of their own market will reward this effort in international markets. However, our study suggests that management should consider a balanced approach to investment expenditure for the development of dynamic capabilities by area. A company that does innovate effectively must be aware that if it does not balance the degree of investment between areas for the creation of dynamic capabilities, its chances of succeeding in international markets in times of economic crisis will be diminished. According to the results of our model, if this investment is not made in developing dynamic marketing capabilities, its competitive advantage in innovation will not be decisive in international results. Executives and boards of directors must, as we have indicated above, make an effort to look beyond the crisis itself, managing these resources in an appropriate and orderly manner among learning, innovation and marketing capabilities, knowing that if the development of any of them is low, it is very likely that the others will be diminished, and therefore the overall results, which, in the case of international markets in periods of crisis, will not be as merciless as in boom periods, and the company's entire operations could be seriously affected. This is why companies should establish performance analyses of their different resources. These analyses would have a long-term orientation, determining whether there is indeed a medium-term equilibrium in their utilization. Knowing this fact, when the environment becomes more hostile and in economic recession, the company could restructure its resources in a more efficient and profitable way, saving time in the movement and management of resources. In this case, the advantage would come from the prior knowledge of the relationship of some resources with others, necessary to make quick and effective decisions on business movements.

In the same way that we dealt with the allocation of expenses, managers must also consider the allocation of personnel among the different areas. Our study, according to the percentage of variance explained, indicates that in periods of economic crisis, the development of the marketing capabilities area is essential to shape learning and innovative processes. Therefore, the manager must carry out a personnel function study to balance this situation, knowing in advance how the capabilities are related. We understand that these are times of scarcity of resources, and even more so of personnel, which in the economic crisis is the first factor to be dispensed with, so the company's management must be more active in terms of harnessing the talent of the people involved in the process, transferring personnel between areas to enhance capabilities. Knowledge of the company's marketing potential in international environments can be a determining factor in overcoming the economic crisis period. The results show that a better marketing performance can be a lever to support the rest of the dynamic capabilities.

Finally, our work also highlights the fundamental importance of marketing from an international business management perspective, in the form of dynamic capabilities, as a factor of cohesion and development for the company and its results in times of economic crisis. The role it plays in this study enables a relationship between the organization and its commercial management, serving as a bridge in that complicated dividing line sometimes drawn. Without marketing there are no results because learning transformed into innovation is neither useful nor generates wealth if we are not able to convince the market. Therefore, the company must fully rely on its marketing capabilities and, in the adverse circumstances of recession periods, an adequate management of these capabilities, enhancing those especially favorable to the company, is the difference between surviving in a turbulent international environment or not surviving at all.

### 6.3 Social implications

From a social and public management perspective, in recent times, where markets have tended towards unification and increasing size, internationalization has been seen as a necessary process by companies and governments. From the point of view of international prestige, as well as the contribution of wealth to balance the balance of payments, this vision has led governments to invest huge amounts of money in all kinds of agencies and organizations that promote international entrepreneurship and the culture of internationalization among companies. Most of these investments are aimed at supporting the internationalization process in its different phases, promoting the domestic market abroad, and establishing alliances with local partners in later phases. However, this paper attempts to rethink this situation, shifting the focus from the process to the company. Although it is necessary for this process to be well defined and analyzed, our results consider that the analysis of the situation must begin with the company itself, and determine what its capability's structure is, what it does well, where it is best, how it does it and how it is able to adapt to changes. From there, we will be in a better position to offer it if not an internationalization process, at least an adaptation of capabilities that will allow it to develop in international markets according to its competitive advantages, as well as a balance to know what capabilities it has to improve and how to position the resources to do so. By reaching these levels, the company will be in a better position to face the internationalization process, making the best use of resources, as well as discarding those whose dynamic capabilities are not developed or balanced. Therefore, the company can take better advantage of foreign resources than governmental organizations oriented to the promotion of foreign trade through the knowledge and assessment of its own capabilities, thus allocating possible aid to those capabilities for which it is more prepared. The company should work on this aspect prior to the arrival of the economic crisis, whose acceleration of decisions will not allow a clear vision of the process and therefore will mean a higher probability of failure.

The analyses we carried out during the theoretical framework and the empirical verifications of our hypotheses make us relate the development of the proposed dynamic capabilities with regular and sustained exports by companies. Therefore, public administrations, through internationalization promotion agencies and programs, should carry out prior studies on the presence of these capabilities in companies, and even try to promote them, in order to obtain better returns on their aid. The programs could cover not only the internationalization process itself, but also the creation of dynamic skills and capabilities at an earlier stage to help maintain a competitive advantage with respect to foreign investment. On the other hand, reality has shown that periods of declining demand can be gradual or immediate, as was the case after the containment measures taken by the recent global pandemic. So, companies must be prepared and strategically oriented to modify and adapt their resource base to extraordinary circumstances that may arise. Our work indicates that it is possible to improve results in these periods if this resource base is founded on a strategy of dynamic capabilities and a monitoring of the environment that allows taking advantage of its opportunities, despite the negative outlook for demand.

## General conclusion

In times of economic crisis, dynamic capabilities become an important tool to enhance companies' strategic options to obtain good international results, especially when they act in a combined manner and adapt at all times to the changing environment. Dynamic marketing capabilities play a fundamental role in the development of the dynamic process, acting both directly on performance and in combination with other capabilities.

## Limitations and future Directions

The treatment of such a specific and concrete environment, such as that of an economic crisis, means that the generalization of results, despite the model's good disposition, needs to be applied with caution. The idiosyncratic nature of dynamic capabilities requires that they be employed in contexts of successive economic crises, something that could determine novel strategies in these turbulent times of COVID-19.

Likewise, the sample size, although having an important statistical power, would need to be increased in order to make the sectorial groups of companies more consistent, and to determine whether there are significant differences between them. The approach or choice of the dynamic capabilities present in the model responds to an exhaustive theoretical review, which does not prevent them from being approached differently or adding others that are not present. Future research should take these aspects into account.

## Data availability statement

The data that has been used is confidential.

## Ethics approval and consent to participate

We have had the consent of the companies to carry out the database, complying with the ethical directives of the University of Seville. The companies gave their informed consent.

## CRediT authorship contribution statement

**Pablo Ledesma-Chaves:** Writing – review & editing, Writing – original draft, Visualization, Validation, Supervision, Software, Resources, Project administration, Methodology, Investigation, Funding acquisition, Formal analysis, Data curation, Conceptualization. **Jorge Arenas-Gaitán:** Writing – original draft, Validation, Supervision, Software, Methodology, Investigation, Formal analysis, Data curation, Conceptualization.

## Declaration of competing interest

The authors declare that they have no known competing financial interests or personal relationships that could have appeared to influence the work reported in this paper.
