# Supplementary material for: Dynamic capabilities in times of economic crisis. A vision of success in international markets
Source: Heliyon. 2023 Nov 22;9(12):e22703. doi: 10.1016/j.heliyon.2023.e22703 (PMC10709518; doi:10.1016/j.heliyon.2023.e22703)
Supplement: Multimedia component 1 [file mmc1.docx]

Research questionnaire. A 7-point Likert scale was used to measure the variables.

| **Dynamic innovation capability** |
| --- |
| Relative to our competitors, my company is good at setting up new types of manufacturing facilities and operations |
| Relative to our competitors, my company is good at learning about technology it has not used before |
| Relative to our competitors, my company is good at recruiting engineers in technical areas it is not familiar with |
| Relative to our competitors, my company is good at assessing the feasibility of new technologies. |
| Relative to our competitors, my company is good at identifying promising new technologies. |
| Relative to our competitors, my company is good at implementing new types of production processes |
| **Dynamic marketing capability** |
| Relative to our competitors, my company is good at assessing the potential of new markets. |
| Relative to our competitors, my company is good at building relationships in new markets. |
| Relative to our competitors, my company is good at setting up new distribution channels |
| Relative to our competitors, my company is good at setting up a new sales force |
| Relative to our competitors, my company is good at leveraging its brand reputation or company image to new markets |
| Relative to our competitors, my company is good at researching new competitors and new customers. |
| Relative to our competitors, my company is good at developing new advertising or promotion strategies |
| Relative to our competitors, my company is good at developing new pricing strategies |
| **Dynamic learning capability** |
| We have a comprehensive program for employee learning |
| We have an organization-wide training and development process, including career path planning, for all our employees |
| Employee learning is a topic that is discussed intensively by the top management |
| The attitude prevailing here is that employee learning in an investment, not an expense |
| We always upgrade employees’ knowledge and skills profiles |
| Managers agree that our organization’s ability to learn is the key to our competitive  advantage |
| **International performance** |
| Rate your organization relative to all other competitors in your principal served market segment (PSMS) over the past year on market share |
| Rate your organization relative to all other competitors in your principal served market segment (PSMS) over the past year on export propensity |
| Rate your organization relative to all other competitors in your principal served market segment (PSMS) over the past year on profitability |
| Rate your organization relative to all other competitors in your principal served market segment (PSMS) over the past year on sales |
